# Correlation of trans fatty acids with the severity of coronary artery disease lesions

**DOI:** 10.1186/s12944-018-0699-3

**Published:** 2018-03-15

**Authors:** Samia Hadj Ahmed, Wafa Kharroubi, Nadia Kaoubaa, Amira Zarrouk, Fathi Batbout, Habib Gamra, Mohamed Fadhel Najjar, Gérard Lizard, Isabelle Hininger-Favier, Mohamed Hammami

**Affiliations:** 1 Research Laboratory LR12ES05 LR-NAFS ‘Nutrition - Functional Food & Vascular Health’ Faculty of Medicine, University of Monastir, Avicene st, 5019 Monastir, Tunisia; 2 Department of Cardiology, CHU Fattouma Bourguiba, Monastir, Tunisia; 3 Department of Biochemistry, CHU Fattouma Bourguiba, Monastir, Tunisia; 4 Team ‘Biochemistry of the Peroxisome, Inflammation and Lipid Metabolism’ EA 7270 / INSERM, University of Bourgogne Franche-Comté, Dijon, France; 5 Laboratory of Fundamental and Applied Bioenergetic, INSERM, Grenoble Alpes University, F-38041 Grenoble, France

**Keywords:** Elaidic acid, Trans C18:2 isomers, Oxidative stress, Lipid peroxidation, Coronary artery disease

## Abstract

**Background:**

Nutritional choices, which include the source of dietary fatty acids (FA), have an important significant impact on coronary artery disease (CAD). We aimed to determine on patients with CAD the relationships between Trans fatty acids (Trans FA) and different CAD associated parameters such as inflammatory and oxidative stress parameters in addition to Gensini score as a vascular severity index.

**Methods:**

Fatty acid profiles were established by gas chromatography from 111 CAD patients compared to 120 age-matched control group. Lipid peroxidation biomarkers, oxidative stress, inflammatory parameters and Gensini score were studied.

**Results:**

Our study showed a significant decrease of the antioxidant parameters levels such as erythrocyte glutathione peroxydase (GPx) and superoxide dismutase (SOD) activities, plasma antioxidant status (FRAP) and thiol (SH) groups in CAD patients. On the other hand, catalase activity, conjugated dienes and malondialdehyde were increased. Plasmatic and erythrocyte Trans FA were also increased in CAD patients compared to controls. Furthermore, divergent associations of these Trans FA accumulations were observed with low-density lipoprotein-cholesterol/ high-density lipoprotein-cholesterol (LDL-C/HDL-C) ratio, Apolipoprotein B (ApoB), lipid peroxidation parameters, high-sensitivity C Reactive Protein (hs-CRP), Interleukin 6 (IL-6), tumor necrosis factor alpha (TNF-α) and Gensini score. Especially, elaidic acid (C18:1 trans 9), trans C18:2 isomers and trans 11 eicosanoic acid are correlated with these parameters. Trans FA are also associated with oxidative stress, confirmed by a positive correlation between C20:1 trans 11 and GPx in erythrocytes.

**Conclusions:**

High level of Trans FA was highly associated with the induction of inflammation, oxidative stress and lipoperoxidation which appear to be based on the vascular severity and might be of interest to assess the stage and progression of atherosclerosis. The measurement of these Trans FA would be of great value for the screening of lipid metabolism disorders in CAD patients.

## Background

Coronary artery atherosclerosis remains the leading cause of cardiovascular morbidity and mortality today in the word, and the association of circulating cholesterol or low-density lipoprotein-cholesterol (LDL-c) with atherosclerosis and coronary artery disease is well documented [1]. Moreover, a positive association between elevated circulating LDL levels and Trans fatty acid (Trans FA) intake has been shown in epidemiological studies [2, 3]. Therefore, the potential for dietary Trans FA to alter or promote the relationship, between the LDL atherosclerosis and coronary artery disease is considerate the mechanism for the deleterious effects of Trans FAs on the cardiovascular system.

Indeed, nutritional choices which include the source of dietary fatty acids (FA), have an important significant impact on coronary artery disease (CAD). In fact, different clinical investigations have revealed a significant positive association between CAD and the consumption of Trans FA [4], which has been found in atherosclerotic lesions and adipose tissue of obese and cardiac patients [5]. It can induce their deleterious effects on cardiovascular disease through an atherogenic action.

Trans FA are produced industrially or naturally. Industrially through partial hydrogenation of liquid plant oils in the presence of a metal catalyst, vacuum, and high heat, and naturally in meat and dairy products via bacterial enzymes where ruminant animals biohydrogenate unsaturated FA. Elaidic acid is the major industrially produced Trans FA in the food, while the major ruminant derived Trans FA is vaccenic acid; both share the characteristic of having one double bond in the “*trans*-” rather than “*cis*-” configuration [6].

The aim of the current study was to determine plasmatic and erythrocyte profiles of FA in CAD Tunisians patients and to determine which Trans FA is/are the most correlated with the different parameters associated with CAD like inflammatory factors, oxidative stress parameters, and Gensini score as an index of vascular severity.

## Methods

### Patients

#### Controls

One hundred twenty controls age-matched were recruited at several diabetes screening days. Among these people none had any cardiovascular risk factors except smoking and menopause with a reduced numbers and no medication use.

#### Patients

A total of 111 Tunisian patients consulting for coronary disorders were recruited from F. Bourguiba University Hospital (Monastir, Tunisia), and were clinically examined by coronary angiography at the department of Cardiology. A Gensini score was calculated for all patients in order to estimate the severity of coronary artery disease (CAD).

CAD patients have a clinical history of unstable angina, previous acute coronary syndromes with or without persistent ST-segment elevation in addition to the presence of several risk factors for cardiovascular disease.

The following data were also obtained: age, sex, and the presence of risk factors (cigarette, smoking, hypertension, diabetes mellitusand dyslipidemia Body mass index (BMI) was calculated from the equation: BMI = weight/height^2^ (kg/m^2^). The most common exclusion criteria which prevented recruitment of different patients were: absence of infectious or acute/ chronic inflammatory diseases, known malignancy, absence of acute/chronic renal failure, or hepatic failure, percutaneous coronary intervention and cerebrolvascular accident.

None of the subjects and controls was using an antioxidant therapy, vitamin supplementation or hormonal replacement therapy for the post menopausal women. The Institution Ethics Committee for studies on human subjects approved this study, and informed consent was obtained from all patients and controls before their enrolment.

### Blood samples preparation

Blood samples from fasting patients were collected into EDTA tube. Then, the plasma and RBCs were separated by centrifuged at 3000 rpm for 10 min at 4 °C. Plasma samples were then removed, and RBCs were washed three times in saline solution (0.9% Sodium chloride (NaCl)). After centrifugation, RBCs were stored at − 80 °C, until biochemical analysis, as aliquots of 100 μL of RBC after realizing a counting of red blood cells. RBC average was 450 million RBCs / 100 μL for all samples. Biochemical measurements were carried out according to validated methods. Plasma glucose concentration was evaluated using an enzymatic kit (glucose oxidase, Randox, Antrim, UK), glycosylated hemoglobin (HbA1c) by an exchange microcolumn chromatographic procedure (Biosystems, Barcelona, Spain), lipid parameters were determined as described by Smaoui et al. [7].

### Coronary angiographic data and Gensini score

All coronary angiography results were interpreted for the presence, extent and severity of CAD, by two experienced interventional cardiologists. Patients were considered as CAD patients according to angiographic results (≥ 50% obstruction in ≥1 coronary artery). Gensini score was used to assess the severity of coronary artery disease: it grades narrowing of the lumen of the coronary artery and scores it as 1 for 1–25% narrowing, 2 for 26–50% narrowing, 4 for 51–75%, 8 for 76–90%, 16 for 91–99% and 32 for a completely occluded artery. This score was then multiplied by a factor according to the importance of the coronary artery. The multiplication factor for a left main stem (LMS) lesion was 5. It was 2.5 for proximal left anterior descending artery (LAD) and proximal circumflex artery (CX) lesions, 1.5 for a mid-LAD lesion, and 1 for distal LAD, mid/distal CX and right coronary artery lesions. The multiplication factor for any other branch is 0.5. The points were then added and the total Gensini score of each patient was calculated. The severity of disease was expressed as the sum of the scores for the individual lesions [8].

### Fatty acids extraction for profile analysis

Fatty acids were analyzed as fatty acid methyl esters (FAMEs) by gas chromatography (GC). Total lipids were extracted from the plasma and RBCs as described by Folch et al. by using a chloroform-methanol (2:1. *v*/v) solvent system containing 0.01% butylated hydroxytoluene as the antioxidant and C17:0 as the internal standard [9]. Aliquots of total lipids were converted into methyl esters using 14% methanol-boron trifluoride (BF-3) at 50 °C for 30 min. FAMEs were analyzed in duplicate and 1 μL of each sample was injected into the GC system (Hewlett Packard. Palo Alto. CA, USA) equipped with a flame ionization detector and a polar fused silica capillary column HP-Innowax with cross-linked PEG (Carbowax 20 M: 30 m × 0.25 mm ID and 0.25 μm as film thickness). The oven temperature was programmed to increase from 180 °C to 250 °C at a rate of 10 °C/min and the injector and detector temperatures were 220 °C and 280 °C, respectively. FAMEs were identified by comparing their retention times with those of individual standards. The fatty acid composition was reported as a relative percentage of the total peak area using an HP Chemstation integrator. The percentage of each fatty acid was determined by dividing the peak area of each fatty acid by the total peak area.

### Oxidative stress measurements

#### Plasma thiol (SH) groups

Protein oxidation in the plasma was evaluated by the disappearance of protein thiol groups [10]. Plasma thiols (SH) were assayed in 20 μL of plasma, using 5,5′-dithiobis(2-nitrobenzoic acid (DTNB)) for deriving the thiol groups. The calibration curve was obtained by mixing two stock solutions of N-acetyl cystein (NAC) in the range of 0.125–0.6 mmol/L. Standards and plasma samples were measured spectrophotometrically at 415 nm (Hitachi 912, B Braun Science Tec, France) in the presence of a phosphate buffer 50 mM, EDTA 100 mM, pH 8 and bis-5,5′-dithio-bis (2-nitrobenzoic acid) 10 mM.

#### Plasma antioxidant status

Plasma antioxidant status was evaluated using ferric reducing ability power (FRAP) assay. The FRAP assay uses antioxidants as reductants in a redox-linked colorimetric method. In this assay, at low pH, a ferric-tripyridyltriazine complex is reduced to the ferrous form, which is blue and monitored by measuring the change in absorption at 593 nm. The change in absorbance is directly proportional to the reducing power of the electron-donating antioxidants present in plasma. The absorbance change is translated into a FRAP value (in μM/L) by relating the change of absorbance at 593 nm of test sample to that of a standard solution of known FRAP value [11].

#### Antioxidant enzymes

Superoxide dismutases (SODs) constitute the primary enzymatic defense against the damage caused by the toxic superoxide anion radical O_2_^.-^ and its derivatives such as peroxynitrite, by catalyzing its dismutation intohydrogen peroxide and oxygen [12]. Erythrocyte copper/zinc superoxide dismutase (Cu/Zn SOD) activity was measured after hemoglobin precipitation by monitoring the auto-oxidation of pyrogallol by the method of Marklund and Marklund [13]. The pyrogallol assay based on the competition between pyrogallol oxidation by O_2_^.-^ and superoxide dismutation by SOD.

The rate of auto-oxidation is taken from the increase in the absorbance at 420 nm. One unit of SOD activity is defined as the amount of the enzyme required to inhibit the rate of pyrogallol auto-oxidation by 50%.

Glutathione peroxidase (GPx) activity, which is a seleno-enzyme involved in protection against hydrogen peroxide (H_2_O_2_) was evaluated in plasma and erythrocyte samples by the modified method of Gunzler [14] using terbutyl hydroperoxide (Sigma Chemical Co, Via Coger, Paris, France) as a substrate instead of hydrogen peroxide. The principle of the assay is based on the coupled reaction with glutathione reductase (GR). Oxidized glutathione (GSSG), produced upon reduction of an organic hydroperoxide by GPx with glutathione (GSH) as electron donor, is recycled to its reduced state by GR and NADPH. The oxidation of NADPH to NADP+ is accompanied by a decrease in absorbance at 340 nm. The rate of decrease in the A340 is directly proportional to the GPx activity in the sample. The assay was performed at 25 °C and pH 7.0 that allowed a stable concentration of GSH in the reaction medium.

#### Determination of lipid peroxidation products

Lipid peroxidation was determined indirectly by measuring the production of malondialdehyde (MDA) in the RBC lysate following the method of Yoshioka et al. [15]. Briefly, 250 μL of RBCs lysate was mixed with 1.25 mL of trichloracetic acid (20%) to precipitate the proteins. Thiobarbituric acid (0.67%) was then added, and the mixture was incubated for 30 min at 95 °C. After cooling to room temperature, 4 mL of n-butanol were added and absorbance was measured at 530 nm. Conjugated dienes (CD) are another indicator of lipid peroxidation and these were measured as described by Esterbauer et al. [16]. The results were expressed as μmoles hydroperoxide / mg protein.

### Inflammatory markers measurements

The determination of TNF-α and IL-6 was measured with a commercial Enzyme-linked immunosorbent assay ELISA kit. Plasma TNF-α and IL-6 levels were determined following the manufacturer’s instructions and were accomplished using the eBioscience kit (San Diego, CA, USA). Hs-CRP levels was determined using an immunonephelometric method (Behring N Latex C Reactive Protein Mono-Analyzer; Behring Diagnostic, Marburg, Germany). In fact polystyrene particles coated with monoclonal antibodies specific to human CRP are aggregated when mixed with samples containing CRP. These aggregates scatter a beam of light passed through the sample. The intensity of the scattered light is proportional to the concentration of the relevant protein in the sample. The results were evaluated by comparison with a standard of a known concentration.

### Statistical analyses

The lipid profile data were analyzed using the Statistical Package for Social Sciences (SPSS 18.0 for Windows). Means of all measurements are presented with standard deviations (mean ± SD). A non-parametric Mann-Whitney test was used. The Spearman correlation test was also used to evaluate the relationships between various parameters. Differences with *p* ≤ 0.05 were considered significant.

## Results

### Patient characteristics

The clinical features and biochemical parameters of controls and patients are summarized in Table 1. One hundred eleven patients had angiographically-proven CAD and120 were control subjects. The mean age of control group was 57.10 ± 12.65 years old versus 60.83 ± 9 years old for the group of patients.

**Table 1:** The demographic, clinical and biochemical characteristics of controls and patients

Variables	Controls *n* = 120	CAD group *n* = 111
Age (years)	57.10 ± 12.65	60.83 ± 90**
Current smoking n (%)	14 (12)	38 (34)**
Diabetes n (%)	0 (0)	43 (39)**
High blood pressure n (%)	0 (0)	52 (47)**
Hyperlipidemia n (%)	0 (0)	50 (45)**
BMI (kg/m^2^)	27.73 ± 3.43	29.44 ± 4.28*
Obesity n (%)	0 (0)	46 (41)**
Menopause n (%)	26 (22)	28 (25)
Family history n (%)	0 (0)	24 (22)**
Gensini score	–	28 (13, 52)**
Total cholesterol (mmol/L)	4.80 ± 0.78	4.97 ± 0.99
LDL-C (mmol/L)	3.10 ± 0.81	3.11 ± 0.92
HDL-C (mmol/L)	1.22 ± 0.44	1.01 ± 0.24**
Triglycerides (mmol/L)	1.33 ± 0.38	1.83 ± 0.86**
Hemoglobin A1c (%)	5.24 ± 0.92	8.61 ± 3.06**
Glucose (mmol/L)	5.10 ± 0.80	9.62 ± 4.70**
ApoA1 (g/L)	1.30 ± 0.15	1.12 ± 0.17**
APoB (g/L)	0.60 ± 0.14	0.77 ± 0.24**
Total cholesterol /HDL	4.31 ± 1.45	5.19 ± 1.76**
Triglycerides /HDL	1.23 ± 0.57	1.96 ± 1.09**
LDL/HDL	2.87 ± 1.30	3.30 ± 1.48*
ApoB/ApoA1	0.47 ± 0.13	0.70 ± 0.21**

Data are expressed as n (%) (number of persons, (percent)), mean ± SD (standard deviation) or median (interquartile, range)

*CAD* coronary artery disease, *BMI* body mass index, *LDL-C* low-density lipoprotein-cholesterol, *HDL-C* high-density lipoprotein-cholesterol, *ApoA1* Apolipoprotein A1, *ApoB* Apolipoprotein B

A significant difference between controls and coronary artery disease patients is indicated by *(Mann Whitney Test). *: *p* < 0.05. A significant difference between controls and patients (CAD group) is indicated by *(*p* < 0.05) or **(*p* < 0.001)

Significant differences in age, smoking, diabetes, hypertension (high blood pressure), hyperlipidemia, body mass index (BMI), obesity, menopause, family history, hemoglobin A1c, glucose, ApoA1, Apo B and ratios: total cholesterol/HDL, triglyceride/HDL, LDL/HDL, ApoB/ApoA1, Gensini score were observed between the CAD patients and control group (*p* < 0.01). Moreover, the means serum HDL-C and triglycerides concentrations were more high in CAD group than in control group (*p* < 0.01). However non significant variation in the total cholesterol and LDL-C between CAD patients and controls was observed (Table 1).

### Fatty acids profiles

Table 2 represents plasmatic FA profile. The analysis of the FA composition showed a significantly higher sum of saturated fatty acids (ΣSFA) in the CAD patients than in control group (37.14 ± 3.41 vs 34.01 ± 5.27). The sum of polyunsaturated fatty acids (ΣPUFA) and unsaturated fatty acids (ΣUFA) were significantly lower in the group of patients than in controls (*p* < 0.01). At the opposite, the sum of monounsaturated fatty acids (ΣMUFA) and the sum of trans fatty acids (ΣTrans FA) were significantly higher in CAD patients than in controls. In fact, significantly higher levels of all Trans FA were observed in CAD patients. FA profile of the RBC is given in Table 3. ΣSFA in RBC of CAD patients were significantly greater than those in RBC of controls. However, ΣPUFA and ΣUFA in RBC were significantly lower in the group of patients than in controls (*p* < 0.01, *p* < 0.05 respectively). ΣMUFA and cis FA in CAD patients were similar than those in controls. Moreover ΣTrans FA was significantly higher in CAD patients comparatively to the controls.

**Table 2:** Fatty acid profiles in plasma of controls, and CAD patients determined by gas chromatography

	Controls *n* = 120	CAD patients *n* = 111
Trans Fatty acids		
C16:1 trans 11	0.59 ± 0.17	0.67 ± 0.25*
C18:1 trans 9	0.15 ± 0.14	0.34 ± 0.23**
C18:1 trans 11	0.36 ± 0.20	0.72 ± 0.56**
C18:2 trans 9 trans 12	0.26 ± 0.47	0.41 ± 0.26**
C18:2 trans 9 cis 12	0.19 ± 0.14	0.30 ± 0.18**
C18:2 cis 9 trans 12	0.21 ± 0.12	0.37 ± 0.23**
C20:1 trans 11	0.23 ± 0.31	0.41 ± 0.40**
C18:2 cis 9 trans 11	0.33 ± 0.32	0.42 ± 0.37*
C18:2 trans 10 cis 12	0.34 ± 0.34	0.53 ± 0.38**
Cis Fatty acids		
C16:1 cis 11	1.33 ± 0.31	2.67 ± 0.88**
C18:1 cis 9	13.18 ± 3.71	15.95 ± 3.28**
C18:1 cis 11	2.21 ± 0.73	2.08 ± 0.57
C18:2 cis 9 cis 12	31.88 ± 6.44	20.56 ± 3.37**
C20:1 cis 11	0.80 ± 0.46	0.70 ± 0.37*
Σ Trans FA	2.68 ± 1.07	4.19 ± 1.96**
Σ Cis FA	49.41 ± 5.86	41.97 ± 5.14**
Σ SFA	34.01 ± 5.27	37.14 ± 3.41**
Σ MUFA	20.25 ± 3.48	24.55 ± 3.28**
Σ PUFA	43.05 ± 5.90	34.12 ± 2.93**
Σ UFA	63.30 ± 5.24	58.67 ± 3.60**

Data are expressed as relative values (%): mean % of total FA ± SD from two independent experiments conducted in triplicate for each patients and controls

*Σ Trans FA* sum of trans fatty acids, *Σ cis FA* sum of cis fatty acids, *Σ SFA* sum of saturated fatty acids, *Σ UFA* sum of unsaturated fatty acids, *Σ PUFA* sum of polyunsaturated fatty acids, *Σ MUFA* sum of monounsaturated fatty acids

A significant difference between controls and patients is indicated by *(*p* < 0.05) and **: *p* < 0.001. The Mann-Whitney test was used

**Table 3:** Fatty acid profiles in red blood cells (RBCs) of controls and CAD patients determined by gas chromatography

	Controls *n* = 120	CAD patients *n* = 111
Trans Fatty acids		
C16:1 trans 11	0.42 ± 0.25	0.62 ± 0.34 **
C18:1 trans 9	0.84 ± 0.37	1.30 ± 0.68**
C18:1 trans 11	1.63 ± 0.55	1.51 ± 0.55
C18:2 trans 9 trans 12	0.50 ± 0.35	0.72 ± 0.43**
C18:2 trans 9 cis 12	0.60 ± 0.50	0.65 ± 0.37*
C18:2 cis 9 trans 12	0.75 ± 0.46	0.54 ± 0.42**
C20:1 trans 11	0.51 ± 0.33	0.57 ± 0.32
C18:2 cis 9 trans 11	0.68 ± 0.40	0.72 ± 0.42
C18:2 trans 10 cis 12	0.97 ± 0.45	0.95 ± 0.51
Cis Fatty acids		
C16:1 cis 11	0.86 ± 0.43	1.15 ± 6.24**
C18:1 cis 9	22.09 ± 5.85	22.67 ± 4.74
C18:1 cis 11	1.73 ± 0.63	1.97 ± 0.58*
C18:2 cis 9 cis 12	9.98 ± 3.19	7.69 ± 2.30**
C20:1 cis 11	0.61 ± 0.30	0.62 ± 0.32
Σ Trans FA	6.91 ± 1.82	7.60 ± 1.92**
Σ Cis FA	35.29 ± 6.41	34.09 ± 5.33
Σ SFA	40.66 ± 5.06	43.17 ± 4.03**
Σ MUFA	32.04 ± 5.83	32.98 ± 4.60
Σ PUFA	20.55 ± 3.59	17.20 ± 2.53**
Σ UFA	52.60 ± 5.90	50.18 ± 4.70*

Data are expressed as relative values (%): mean % of total FA ± SD from two independent experiments conducted in triplicate for each CAD patients and controls

*Σ Trans FA* sum of trans fatty acids, *Σ cis FA* sum of cis fatty acids

A significant difference between controls and patients is indicated by *(*p* < 0.05) and **: *p* < 0.001. The Mann-Whitney test was used

**Fig. 1:**
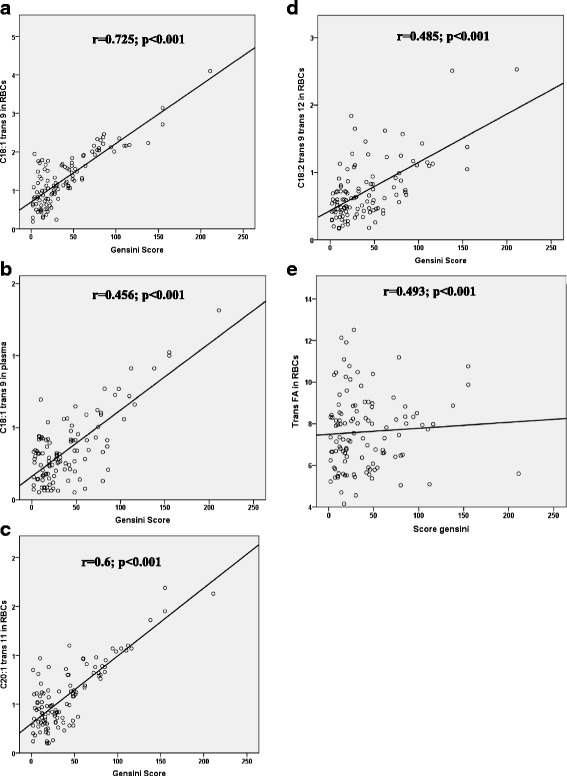
Evaluation of the relationships between different trans fatty acid levels in red blood cells and plasma, and Gensini score. The relationships between the levels of: **a** C18:1 trans 9 in red blood cells (RBCs) and **b** plasma, **c** C20:1 trans 11 level in RBCs, **d** C18:2 trans 9 trans 12 in RBCs and **e** Trans fatty acids (Trans FA) (expressed as relative values (%)) and Gensini score was determined. The Spearman correlation test was used to evaluate the relationships between these parameters. A positive correlation was found in the group of CAD patients with vascular severity

There was a strong positive correlation between Gensini score and the RBCs levels of ΣTrans FA (*r* = 0.49, *p* < 0.001) as well as between Gensini score and RBCs levels of C18:1trans 9, C18:2 trans 9, trans 12), C20:1trans11 and plasma level of C18:1 trans9 (*r* = 0.7, *r* = 0.48, *r* = 0.6 *r* = 0.4, *p* < 0.001 for all, respectively) (Fig. 1).

### Evaluation of anti-oxidative stress parameters in plasma and red blood cells

Redox state was estimated by plasmatic levels of FRAP and total thiol (-SH) content, as well as RBC catalase, superoxide dismutase (SOD) and glutathione peroxidase (GPx) activities (Table 4). Plasma antioxidant status was evaluated using ferric reducing ability power (FRAP) assay.

**Table 4:** The values of inflammatory markers, anti-oxidative parameters and lipid peroxidation markers in CAD patients and controls

	Controls *n* = 120	CAD patients *n* = 111
Inflammatory markers (plasma)		
TNF-α pg/mL	3.22 ± 3.27	7.40 ± 3.38**
IL-6 pg/mL	4.39 ± 1.50	7.12 ± 3.90**
hs-CRP (mg/L)	1.22 ± 0.95	3.03 ± 2.26**
Antioxidative parameters		
FRAP μMol/L (plasma)	905.99 ± 216.76	842.00 ± 171.15*
Thiol SH μMol/L (plasma)	308.70 ± 55.04	172.13 ± 65.55**
Catalase (U/g HB) (RBCs)	73.83 ± 59.82	299.55 ± 11.00**
GPX (U/g HB) (RBCs)	41.51 ± 9.70	32.55 ± 5.97**
SOD U/g HB (RBCs)	2103.26 ± 286.12	1531.66 ± 424.55**
Lipid peroxidation markers (RBCs)		
MDA (nM/gP) (RBCs)	12.80 ± 3.42	18.01 ± 5.16**
Conjugated diene (nM hydroperoxides / gP) (RBCs)	111.10 ± 53.46	155.99 ± 80.07**

Data are presented as mean ± SD. Inflammatory markers (TNF-α, IL-6), anti-oxidative parameters (FRAP: ferric reducing ability of plasma

*THIOL SH* Plasma protein thiols (-SH), *GPX* Glutathione peroxidase, *SOD* Superoxyde dismutases) and lipid peroxidation markers (Conjugated dienes and MDA: Malondialdehyde) levels are shown in the RBCs of controls and patients, *HB* hemoglobin

Statistical analyses were performed with the Mann-Whitney test. Significant differences between controls and CAD patients were observed as follows; *: *p* < 0.05; **: *p* < 0.001. Two independent experiments conducted in triplicate for each CAD patients and controls were realized

Plasma antioxidant status estimated by FRAP was significantly decreased in the group of CAD patients in comparison with control (842 ± 171.5 vs 905.99 ± 216.76, respectively). The levels of reduced plasmatic thiol (-SH) in CAD patients was significantly decreased compared to control (*p* < 0.01). Superoxide dismutase activity in RBCs of CAD patients was significantly increased (*p* < 0.01). Opposite results, significant decreased of GPx activity in RBC, were found for patients (Table 4). A positive correlation is observed between the GPx activity in RBC and C20:1trans 11 (*r* = 0.22; *p* < 0.05) (Table 5).

**Table 5:** Evaluation of the relationships between Trans FA, GPx and inflammatory response parameters (IL-6, TNF-α and hs-CRP) in RBC and plasma

	GPx	IL-6	TNF-α	hs-CPR
Trans FA in plasma				
C 16:1 trans 11	ns	r=0.28 ( *p*<0.05)	ns	ns
C18:1trans 9	ns	r=0.55 (*p*<0.001)	ns	r=0.30 (*p*<0.001)
C20:1trans 11	r=0.22 (*p*<0.05)	r=0.27 (*p*<0.05 )	ns	ns
C18:2 trans 9,trans 12	ns	ns	r=0.30 (*p*=0.02)	ns
C18:2 trans 10, cis 12	ns	ns	r=0.30 (*p*=0.03)	ns
Trans FA in RBC				
C18:1 trans 9	ns	r=0.46 (*p*<0.001)	ns	r=0.20 (*p*<0.05)
C20:1 trans 11	ns	r=0.60 (*p*<0.001)	ns	r=0.35 (*p*<0.001)
C18: 2 trans 9	ns	ns	ns	ns
∑Trans FA	ns	r=0.37 (*p*=0.004)	ns	ns

r: spearman correlation. Significant differences between controls and CAD patients were observed as follows (*p*<0.05; *p*<0.001). ns: no significant

### Evaluation of lipid peroxidation biomarkers in red blood cells

The results of the quantification of various RBC lipid peroxidation products of patients and controls are showed in Table 4.

**Fig. 2:**
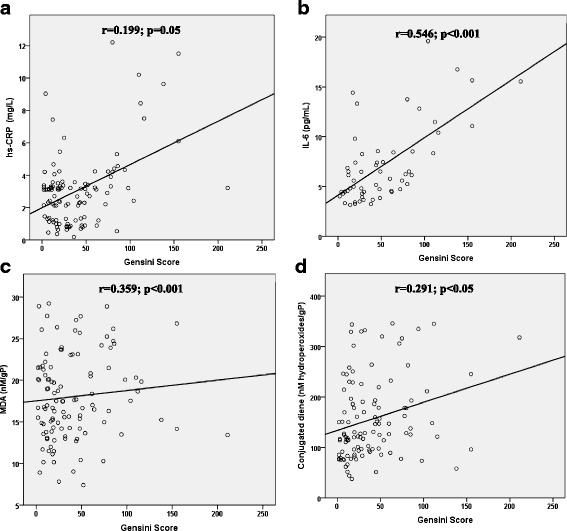
Evaluation of the relationships between lipid peroxydation, inflammatory response parameters, and Gensini score. The relationships between the levels of: **a** hs-CRP (in plasma), **b** IL-6 (in plasma), **c** MDA and **d** conjugated dienes in red blood cells (RBCs) (expressed as mean ± SD) and Gensini score was determined. The Spearman correlation test was used to evaluate the relationships between these parameters. A positive correlation was found in the group of CAD patients with vascular severity

Significantly higher levels of MDA and CD were observed in RBC of CAD patients than controls (*p* < 0.01). These lipid peroxidation parameters were correlated positively with the vascular severity estimated by Gensini score (*r* = 0.359; *p* < 0.001 for MDA and *r* = 0.291; *p* < 0.05 for CD) (Fig. 2).

**Fig. 3:**
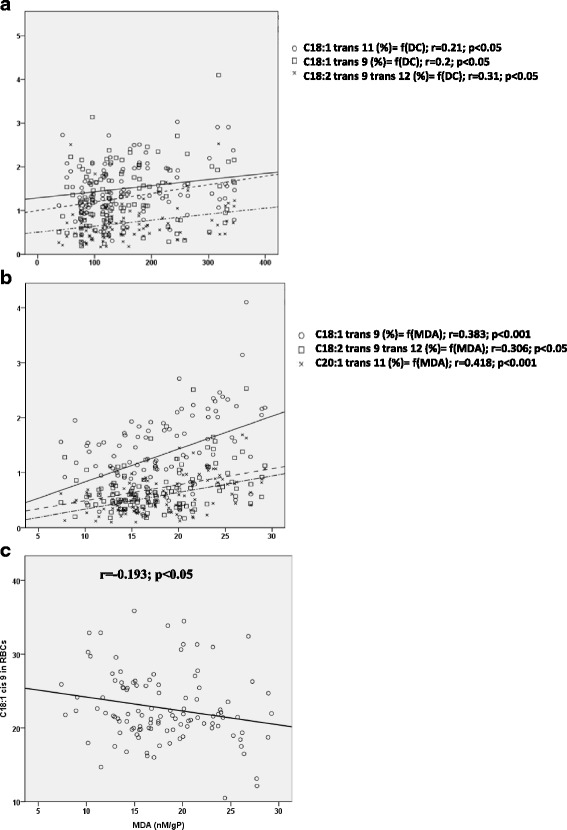
Evaluation of the relationships between trans fatty acids and lipid peroxidation markers. **a** Correlation in red blood cells (RBCs) between C18:1 trans 11, C18:1 trans 9 and the C18:2 trans 9 trans 12 levels and conjugated dienes (CD) in CAD group. **b** correlation in RBCs between C18:1 trans 9, C18:2 trans 9 trans 12 and C20:1 trans 11 and malondialdehyde (MDA). **c** Correlation in RBCs between C18:1 cis 9 and malondialdehyde (MDA). The Spearman correlation test was used to evaluate the relationships between these various parameters. Fatty acids are expressed as relative values (%)

Interestingly, in CAD patients we performed a significant positive correlation between CD and levels of C18:1 trans 11, C18:1 trans 9 and C18:2 trans 9, trans 12 in RBC (*r* = 0.21, *r* = 0.2 and *r* = 0.3 respectively, *p* < 0.05) (Fig. 3a).

A significant positive correlation was found in RBCs of CAD patients between MDA and levels of some Trans FA such as C18:1 trans 9, C18:2 trans 9, trans 12 and C20:1 trans 11 (*r* = 0.4, *p* < 0.001; *r* = 0.3, *p* < 0.05 and *r* = 0.42, *p* < 0.001, respectively) (Fig. 3b). In opposite, a significant negative correlation was noted in RBC of patients between MDA and the level of C18:1cis 9 (*p* < 0.05) (Fig. 3c).

### Inflammatory markers measurements

Table 4 shows that patients had higher plasma levels of all inflammatory markers. In fact, a very significant difference was observed in TNF-α, IL-6 and hs-CRP in CAD patients compared to controls (*p* < 0.001). In addition, hs-CRP and IL-6 were positively correlated with Gensini score (*r* = 0.199; *p* = 0.05 and *r* = 0.546; *p* < 0.001, respectively) (Fig. 2).

### Evaluation of the relationships between plasmatic and red blood cells levels of trans fatty acid and inflammatory factors

In CAD patients, hs-CRP levels were significantly correlated with the RBC levels of C18:1 trans 9 (*r* = 0.2, *p* < 0.05). Circulating hs-CRP levels exhibited a strong positive correlation with the plasma level of C18:1trans9 (*r* = 0.3, *p* < 0.001) as well as with the RBC level of C20:1 trans 11 (*r* = 0.357, *p* < 0.001) (Table 5).

Plasma Il-6 levels of CAD patient were significantly correlated with many Trans FA including RBC and plasma levels of C18:1 trans 9 (*r* = 0.46, *r* = 0.55, *p* < 0.001 respectively) and C20: trans 11 (*r* = 0.6, *p* < 0.001; *r* = 0.27, *p* < 0.05 respectively) as well as the plasma levels of C16:1 trans 11 (*r* = 0.28, *p* < 0.05) and the RBCs Trans FA (*r* = 0.37, *p* = 0.004) (Table 5).

A significant associations were noted in CAD patients between the plasma TNF-α levels and plasma levels of C18:2 trans 9, trans 12 (*r* = 0.3, *p* = 0.02) and C18:2 (trans 10, cis 12) (*r* = 0.3, *p* = 0.03) (Table 5).

## Discussion

The risk of atherosclerotic cardiovascular disease is largely linked to modifiable environmental factors, including diet. In our study, we demonstrated that excessive intake of Trans FA might accelerate atherosclerotic lesion formation, inflammatory cytokines and oxidative stress induction. The adverse effects of Trans FA have been generally explained by worsening plasma lipid profiles.

In fact, the comparison between patients and healthy subjects was performed. As expected TG, ApoB and ApoB/ApoA1 ratio levels were found higher in CAD patients than controls, while, HDL-C and ApoA1 levels in CAD patients were lower compared to controls. No significant differences between patients and controls in cholesterol, and LDL-C were observed. However, a very significant increase of Trans FA was found in CAD patients compared to controls which can might has an effect on the alteration of these parameters.

In previous studies, it has been demonstrated that dietary Trans FA increased in mice plasma levels of triglyceride but did not affect cholesterol and LDL-C plasma levels [17, 18]. The reason for the lack of the LDL raising effect by Trans FA in mice remains unclear, but it may be explained by the species difference of lipid metabolism. It has been showed that Trans FA has different direct adverse effects on the vessel wall beyond its impact on plasma lipid profile [19].

Many reports attribute more weight to the ApoB/ApoA1 ratio as an index of cardiovascular risk [20] and it is considered as an effective predictor of CAD risk in overweight and obesity [21]. Therefore, ApoB/ApoA1 levels were better than LDL-C levels in predicting the risk of CAD. The higher level of the ApoB/ApoA1 ratio is, the more likely cholesterol is to be deposited in the arterial wall, thereby provoking atherogenesis [22].

Moreover, in RBC, we showed a positive correlation between the ApoB and C18:1trans 11 and C18:2 trans 9, trans 12. However, a negative correlation is noted between ApoB and ∑PUFA. This result might confirm the important role played by Trans FA on the alteration of lipid profile, supporting that Trans FA worsens quality of the plasma lipids.

Inflammation is an important contributor to atherosclerosis. High levels of inflammatory markers are associated with atherosclerotic disease. CAD patients showed increased plasma levels of inflammatory markers, including IL-6, TNFα and hs-CRP C. Our results are in agreement with previous findings [23], in demonstrating that plasma levels of several mediators of inflammation (eg, IL-6, TNFα and hs-CRP) are increased in CAD patients compared to controls. Moreover a positive correlation is noted between the vascular severity index and inflammatory biomarkers (IL-6, hs-CRP).

The positive correlation between Trans FA and hs-CRP, IL-6 and TNF-α observed in this study, let’s us presume that the high level of Trans FA was associated not only with lipid, but also with the induction of inflammation and in consequence the atherosclerosis disease confirmed by the positive correlations between inflammatory markers (IL-6, h-CRP), vascular severity (Gensini score) and Trans FA. In fact, an excessive intake of Trans FA has been reported to be associated with systemic inflammation and endothelial dysfunction [24].

Our results are in accordance with the study of Mozaffarian et al. [25] in which a positive correlation was observed between intake of Trans FA and two markers of systemic inflammation which are soluble tumor necrosis factor receptor 1 and receptor 2 (TNF-R1 and TNF-R2). This positive correlation was seen for both total trans, 18:1 trans and 18:2 trans. Furthermore, in women with higher body mass index, a significant positive correlation with levels of IL-6 and CRP as markers of systemic inflammation was observed [25].

These pro-inflammatory effects which might be due to Trans FA accumulation can account at least in part for the pathogenesis of the atherosclerotic lesion development.

In this study, a search for markers of oxidative stress was conducted both in plasma and erythrocytes. The use of erythrocytes is complementary to the plasma as it provides information about the effects of the environment on the plasma membrane. The plasma and erythrocyte approach revealed a strong oxidant stress in coronary. Thus, in erythrocytes, GPx and SOD activities decreased whereas catalase activity, conjugated dienes (CD) and MDA increased. In plasma, antioxidant capacity estimated by FRAP assay and the rate of protein thiols-SH groups decreased significantly. These lipid (lipid peroxidation) and protein alterations (protein thiols SH-groups) is in agreement with the decreased of enzymatic antioxidant defenses (SOD and GPx). Furthermore, the observed increase in erythrocyte Cu,Zn-SOD, associated to a decrease in GPx, leading to a higher risk of H_2_O_2_ accumulation, is a common feature of the route toward type 2 diabetes mellitus [26], ageing [27], and neurodegenerative diseases like a Down syndrome due to a overexpression of SOD without increased in GPx [28]. The increased catalase which act at the peroxisome level on to catalyzed peroxide is an adaptative but insufficient response to cope with overproduction of ROS (superoxide anion and hydrogen peroxide).

It has been reported that Trans FA can modulate the susceptibility of oxidative stress, due to changes of FA composition in cell membrane [25].

It has been shown that oxidative stress contributes to worse coronary patient outcomes [29, 30]. This result was confirmed by the presence of reactive lipids such as malondialdehyde and HNE in addition to the presence of antioxidant enzymes such as catalase [31].

The results therefore underline that oxidative stress is not only stimulated but it is powerful and deleterious in coronary artery diseases. Moreover, some markers of oxidative stress appear to be based on the vascular severity and might be of interest to assess the stage and progression of atherosclerosis.

Thus the levels of MDA and CD in erythrocytes, more important in the later stages of the atherosclerosis, could constitute a biomarkers used to apply an antioxidant timely treatment to prevent lipid peroxidation and therefore the impact on cellular functions can be irreversible and deleterious.

Divergent associations of different circulating Trans FA subtypes was found with Gensini score, LDL/HDL-C ratio, ApoB, lipid peroxidation parameters (MDA and CD), GPx, hs-CRP, IL-6 and TNF-α.

The Trans FA correlated with those parameters are especially: C18:1trans 9 (elaidic acid) trans C18:2 isomers (trans isomers of linoleic acid) and C20:1trans11 (trans 11 eicosanoic acid). It was earlier shown that trans configuration of FA with one or more bonds raise the LDL / HDL cholesterol ratio (especially C18:1trans 9 and C20:1 trans isomers) [32], which noted in our study, and therefore, presumably, the risk of CAD. As well, trans C18:2 isomers were most adversely associated with total mortality, mainly due to the increased risk of CAD [33]. In fact, it has been shown that the level of Trans FA accumulation in atherosclerotic plaques is around 0.1% and that elaidic acid is the main trans isomer in plaques [34]. In addition to several possible mechanisms that mediate the association between Trans FA and CAD, it was shown that Trans FA promotes thrombogenesis [35] and inhibits the conversion of linoleic acid to arachidonic acid and to other n-6 PUFA. This activity disturbs and impairs FA metabolism and causes major changes in FA composition in the aorta in association with activation of the systemic inflammatory response [35]. Mensink and Katan showed that it was difficult for macrophages to transform elaidic acid because trans-unsaturated FA are rapidly incorporated into new cell membranes after their consumption [36].

In the study of Siguel et Lerman [37], plasma total trans, C16:1 trans and also C18:2 trans fatty acids were significantly higher in persons with CAD as compared with controls. Levels of individual Trans FA in platelets were correlated with the extent of an angiographically assessed CAD score [38]. In addition, and after adjustment for established CAD risk factors, C18:1 trans 9 is positively correlated with the extent score. However, there was no correlation with trans vaccenic acid and other Trans FA [38]. Regarding to our study, a positive correlation between the index of vascular severity estimated with Gensini score and C18:1 trans 9 (in RBC and in plasma), erythrocyte C18:2 trans 9 trans 12 and C20:1 trans 11 was observed. These results suggest that these Trans FA positively contribute to the severity of vascular complications.

In addition, high levels of Trans FA may represent a direct source of oxidative stress for the organism [39]. In agreement, in our study the high level of Trans FA showed, an association with stress oxidative, confirmed by a positive correlation between C20:1 trans 11 and GPx in RBC (*r* = 0.22, *p* < 0.05).

Besides, the impact of oxidative events on Trans FA generation remains to be determined. It has been suggested that free radicals formed during stressful conditions are able to induce the formation of Trans FA *in vivo* via cis-trans isomerization of their cis precursor [40]. Also, it is plausible to speculate that the depletion of ∑cis FA and the increase of ∑Trans FA result in peroxidation of unsaturated FA, leading to the degradation of phospholipids and ultimately result in cellular deterioration [41] which explain the increase in the MDA and DC levels in our study. This deduction is supported by the positive correlation between the both parameters of lipid peroxidation (CD and MDA) and some trans FA (C18:1 trans 11, C18:1 trans 9 and the C18:2 trans 9 trans 12) and in addition between MDA and C20:1 trans 11. In the other hand a negative correlation is established between MDA and oleic acid (C18:1 cis 9). This result was expected as oleic acid is a well-documented fatty acid associated with a lower risk of cardiovascular disease as a part of the Mediterranean diet profile [42].

Despite that plasma vaccenic acid C18: 1 trans 11, as a ruminant Trans FA, was significantly higher in CAD patients in comparison to controls and despite that no changes were observed in erythrocytes, which reflect dietary habits of recent weeks and months, however, no correlation was observed nor with vascular severity neither with the parameters of inflammation and oxidative stress.

In fact, to date, there is no conclusive evidence supporting that consumption of ruminant Trans FA especially C18:1 trans 11 is related to adverse physiological effects [43]. Epidemiological, clinical and in vitro studies suggest that the intake of C18:1 trans 11 has no relationship to coronary heart disease or inflammation. Furthermore, this Trans FA may impart health benefits due to its function as a dietary precursor of cis 9, trans 11-CLA [44].

In humans, vaccenic acid is a precursor of the conjugated linoleic acid (CLA) C18:2, cis 9, trans 11 [45]. In fact, in the study of Terpstra [46] regarding the effect of CLA on plasma lipids in humans, showed that supplementation with a chemically prepared mixture of CLA isomers hasn’t an effect on plasma lipids but may have distinct biological effects. In contrast, metabolic trials have shown that industrially C18:1 trans isomers can affect unfavorably the plasma lipid profile [19].

Our findings support further experimental investigation of how Trans FA might affect cell membrane and ion channel functions. Specific Trans FA appear to be preferentially incorporated into lipid rafts or caveolae that modulate membrane receptor function [47]. In addition, our findings are in keeping with our previous results [48], which showed that the toxic effect of elaidic acid in human monocytes is essentially reflected in their ability to induce inflammatory responses mediated by TNF- α and MCP-1, an increase in superoxide anion production, an activation of CD36 and lipid droplet accumulation. All of which induce cell morphology changes and contribute to the transformation of monocytes into foam cells which are a hallmark and characteristic feature of early stage atherosclerotic lesion formation [48].

Inflammatory response induction might be due to the effect of Trans FA as an agonist of the toll-like receptor 4 (TLR4) complex [49], which activates pro-inflammatory pathways and induces cytokine expression in different cell types, promotes macrophage accumulation and then inflammation [50]. However, the high expression of CD36, produced especially with EA, indicates a high uptake of oxidized lipoproteins [51], which consequently contributes to macrophage conversion to foam cells [52].

Contrary to CLA, the other trans C18:2 isomers are known to have several negative effects. It has been suggested that these trans-C18:2 could affect the essential fatty acid metabolism. In fact, the trans-9 trans-12 C18:2 isomer is a competitive inhibitor for the conversion of linoleic to arachidonic acid and a completely blocking C20:5 ω-3 formation [53].

The significant decrease of the antioxidant parameters levels such as GPx, SOD, FRAP and Thiol SH; showed in CAD patients and the positive correlation between the Gensini score and peroxidation parameters (MDA and DC), let’s us presume that CAD are associated with the oxidative stress and the Trans FA which can be considered as the cause and the result of the induction of this oxidative stress.

## Conclusions

In conclusion, from these findings, we deduce that in CAD patients, Trans FA are accumulated abundantly in plasma and RBC, in addition to the induction of inflammation and oxidative stress. It is known that there is complex molecular interaction between inflammation and oxidative stress, and Trans FA seem to be involved in this cross talk. Trans FA seems to have diverse actions on cellular components of the arterial wall.

Trans FA especially elaidic acid (C18:1trans 9), trans isomers of linoleic acid (trans C18:2 isomers) and trans 11 eicosanoic acid (C20:1 trans 11) are correlated with inflammatory parameters (IL-6, hs-CRP and TNF-α), indicators of oxidative stress (MDA and CD) and vascular severity, parameters associated to the development of atherosclerosis.

Taken together, these negative effects of Trans FA, as a whole, are likely to contribute to accelerate and aggravate atherosclerotic lesions. In the present study we provided a novel insight regarding the pro-atherogenic effects of Trans FA in human.

In this context, Trans FA might be considered as an important CAD biomarkers and the measurement of these Trans FA would be of great value for the screening of lipid metabolism disorders in CAD patients.

Despite these negative effects, in Tunisia, no serious study regarding of the consumption of Trans FA currently exists. However, due to the impressive variety of lipid structures that can be generated, it is to expect that a National reflection on the safety and quality control of hydrogenated oils consumed in Tunisia and their impact on health must be imposed in the future.

International recommendations concerning the maximum daily Trans FA intake still scarce. However, due to the detrimental health effects of industrial Trans FA several proposals to keep the Trans FA intake as low as possible [54] and several countries introduced legislation that enforces a mandatory declaration of Trans FA content on food labels.

## Abbreviations

ApoA1: Apolipoprotein A1
ApoB: Apolipoprotein B
BMI: Body mass index
CAD: Coronary artery disease
CD: Conjugated dienes
Cu/Zn SOD: Copper/zinc superoxide dismutase
FA: Fatty acid
FAMEs: Fatty acid methyl esters
FRAP: Ferric reducing ability power
GC: Gas chromatography
GPx: Glutathione peroxydase
GS: Gensini score
HbA1c: Glycosylated hemoglobin
HDL-C: High-density lipoprotein-cholesterol
hs-CRP: High-sensitivity C Reactive Protein
IL-6: Interleukin 6
LDL-C: Low-density lipoprotein-cholesterol
MDA: Malondialdehyde
NaCl: Sodium chloride
RBCs: Red blood cells
SOD: Superoxide dismutase
TNFα: Tumor necrosis factor alpha
Trans FA: Trans fatty acid
Σ cis FA: Sum of cis fatty acids
ΣMUFA: Sum of monounsaturated fatty acids
ΣPUFA: Sum of polyunsaturated fatty acids
ΣSFA: Sum of saturated fatty acids
ΣTrans FA: Sum of trans fatty acids
ΣUFA: Unsaturated fatty acids
